# Dissemination of colorectal cancer information among Hispanic patients and their social network

**DOI:** 10.1186/s12889-024-20095-7

**Published:** 2024-09-27

**Authors:** Linda K. Ko, Sou Hyun Jang, Edgar Rodriguez, Miruna Buta, Genoveva Ibarra, Daniel Reuland

**Affiliations:** 1 Department of Health Systems and Population Health, University of Washington, Seattle, WA USA; 2 Division of Public Health Sciences, Fred Hutchinson Cancer Center, Seattle, WA USA; 3 Department of Sociology, Korea University, 145 Anam-Ro, Anam-Dong, Seongbuk-Gu, Seoul, South Korea; 4 Department of Internal Medicine, University of North Carolina Chapel Hill, 101 East Weaver Street, Campus, Box 7293, Carrboro, NC 27510 USA

**Keywords:** Colorectal cancer screening, Patient decision aids, Hispanic patients, Health communication, Social network

## Abstract

**Background:**

Colorectal cancer (CRC) screening decision aids can inform patients about CRC screening benefits, costs, and procedures. Patients who receive the decision aid report wanting to share the information with their families and friends. We evaluated a CRC screening decision aid on Hispanic patients’ communication to their alters and whether patient-alter communication leads to alters’ CRC screening intention.

**Methods:**

We conducted a one-arm pre/post study of Hispanic patients and their alters; patients (*n* = 42) and their alters (*n* = 19) were recruited from a clinic site in Yakima County, Washington State. Patients viewed a CRC screening decision aid at the clinic site. Survey data from patients and alters were collected via telephone including patients’ communication with their alters about CRC screening after viewing the decision aid and alters’ intention to be screened for CRC after talking to the patient.

**Results:**

Most participants reported sharing CRC information with their alters after viewing the decision aid, and most alters confirmed they had received CRC information from participants (68%). The decision aid was associated with participants' own intention to undergo CRC screening and with alters' intention to be screened for CRC using a fecal occult blood test (*p* = 0.014) and sigmoidoscopy (*p* = 0.011).

**Conclusions:**

Patient decision aids have the potential to increase CRC screening behavior beyond the decision aid recipients to their social network.

**Trial registration:**

Trials Registration Number: NCT04444232 “Retrospectively registered.”

**Supplementary Information:**

The online version contains supplementary material available at 10.1186/s12889-024-20095-7.

## Background

The U.S. Department of Health and Human Services Healthy People 2030 initiative benchmark is for 74.4% colorectal cancer (CRC) screening among adults aged 45 to 75 [1], yet as of 2020, the overall CRC screening rate is 72%, falling short of this benchmark [2]. Furthermore, compared with 74% of non-Hispanic whites, only 64% of Hispanics are up to date with CRC screening guidelines [2]. Due to lower participation rates in CRC screening among Hispanic communities compared to white communities, Hispanic men and women are consequently more prone to being diagnosed at advanced stages of the disease and experiencing worse cancer mortality outcomes, exacerbating health disparities [3–7].

Patient decision aids are interventions designed to inform patients about healthcare decisions and help them overcome barriers related to healthcare communication and decision-making [8–10]. CRC screening decision aids have been created to provide health information about three recommended screening options: fecal occult blood test (FOBT), sigmoidoscopy, and colonoscopy, and they include descriptions of the risks, benefits, costs, and procedures of each test [9–12]. CRC decision aids can lead to better knowledge among patients, intention to participate in screening [13], complete screening tests as well as increase patient-physician communication around CRC [9]. One previous study showed that an overwhelming number of patients (92%) who viewed the decision aid indicated that they would share the information with their families and friends [11]. However, no studies have examined whether information sharing occurs between patients and their social network by gathering both perspectives.

Information processing models illustrate ways that persuasive health communications can influence information processing, change attitudes and behaviors, and lead to information dissemination [14, 15]. The model states that individuals are more likely to disseminate information from cancer communication to their social network if they are exposed to the communication, attend to the information, like the information, and learn from the information [14–17]. Social network refers to the web of social relationships that facilitate social function and exchange of support among network members [16]. Previous research has shown that members of networks characterized by close ties, homogeneity, and reciprocal linkages are likely to exchange more support (e.g., informational support) and exert greater social influence on one another with regard to adoption of norms [16]. Hispanic social networks have been characterized as being close-knit, homogeneous, and reciprocal; thus, members may exert a large amount of social influence on one another [18–20].

Past studies have examined dissemination of CRC communication materials, where researchers tabulated frequencies on the distribution of health materials (e.g., pamphlets, flyers, DVDs) to individuals at a specific site [21–23]. However, scant attention has been paid to understand the dissemination of cancer communication, extending from one individual to another within social networks, and the impact of the communication on network members’ intention to be screened for CRC. This study aims to 1) evaluate the effect of a CRC decision aid on Hispanic patients' CRC screening intentions and their communication with social network members (referred to as “alters”) about the decision aid, and 2) investigate the relationship between patient-alter CRC screening communication and their alters’ intention to screen for CRC.

## Materials and methods

### Study design

We conducted a one-arm pre/post study of Hispanic participants and their alters; participants were patients from a primary care clinic in Yakima County in Washington State. Fifty percent of residents in Yakima County are Hispanics [24], and the collaborating primary care clinic site is the largest clinic in Yakima County. The clinic site self-selected themselves as collaborators after hearing the research team present the study at an annual local network meeting of clinics. Hispanic participants were invited to view the decision aid. Two bilingual (Spanish and English) community health workers assisted with recruitment and data collection. All study procedures were carried out according to an IRB-approved protocol (The Fred Hutchinson Cancer Center, IRB #8182).

### Recruitment

Patients were recruited via the clinic registry. A clinic staff member conducted queries of patient registration data to identify individuals aged 50–75 years old. Bilingual community health workers sent a mailed letter explaining the purpose of the study to the identified individuals. The letter also included the study telephone number to call if potential participants were interested in learning more about study, had questions, or wished to opt-out of the study. Two weeks following the mailed letter, bilingual community health workers conducted follow-up telephone calls to assess eligibility and query interest in participating. Ten separate calls on different dates and times were made to establish contact with a potential participant as suggested by the community health workers. Eligibility criteria included: 1) between ages 50 and 75 years old, based on the Healthy People 2020 guideline at the time of data collection and intervention in 2014–2015; 2) self-identified as “Hispanic” or “Latino;” 3) had no family history of CRC; 4) had no personal history of CRC; 5) had neither an annual stool-based test through fecal occult blood test (FOBT) or fecal immunochemical test (FIT) within the past year, nor a colonoscopy within the last 10 years (deemed not up-to-date with CRC screening by U.S. Preventive Services Task Force recommendations); 6) had at least one alter aged 50–75 years old.

**Fig. 1:**
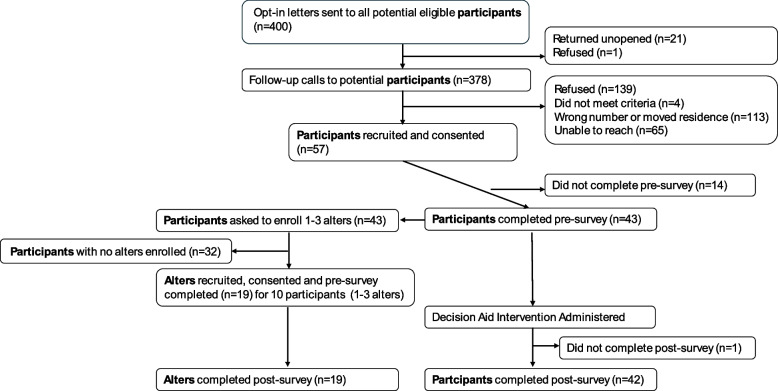
Recruitment and retention flowchart

Once the bilingual community health worker established contact over the phone with a patient who agreed to participate, they obtained their verbal informed consent to participate and administered the pre-survey (*see* Appendix A). Participants who completed the pre-survey (*n* = 43) were instructed to tell their alters to call the study telephone number. 19 alters contacted the community health workers on behalf of 10 participants (1–3 alters per participant). The community health workers enrolled the eligible alters when the alters called the study phone number and agreed to participate in the study. The community health workers obtained the alter’s verbal informed consent to participate and then administered the pre-survey on the phone in English or Spanish. Patients whose alters were enrolled were scheduled to receive the intervention. Figure 1 depicts a recruitment and retention flowchart illustrating the recruitment and retention of participants. We sent 400 mailed letters to potential participants. Twenty-one letters (5.25%) were returned unopened and one potential participant (0.25%) replied to say they did not want to be contacted. We made follow-up calls to 378 (94.50%) patients and recruited 57 patients (14.25%). Among the remaining patients, 139 (34.75%) refused to participate, four (1%) did not meet one of the six eligibility criteria, 113 (28.25%) had a wrong number or had moved residence, and 65 (16.25%) could not be reached after ten attempts. Of those recruited, 43 (75.40%) patients received the interventions, while the remaining 14 (24.56%) patients did not receive the intervention due to loss to follow-up. One patient could not be reached to complete the post-survey, leading to a total of 42 patients. Out of the 42 patients, 32 identified alters, but their alters did not contact the study team. The remaining 10 patients had alters, with each individual having a range of 1 to 3 alters. Patients received a $30 check for completing the pre-survey and viewing the decision aid and a $15 check for completing the post-survey. The alters were mailed a $25 check for their participation in both pre and post surveys.

### Intervention

The decision aid was a previously tested multimedia intervention that included a 14-min English or Spanish language video and a printed brochure [25]. The brochure is included in the Appendix B. The video provided an overview of the rationale for CRC screening, specific information about FOBT (which was the most widely available screening test at that time) and colonoscopy, vignettes from patients about their decision to be screened and why they chose a particular screening test, and a summary of the key characteristics of the two screening tests, including test frequency, cost, overall effectiveness, time required, discomfort, and risk of complications. At the end of the video, patients were prompted to select one of three pre-printed, color brochures corresponding to their readiness for screening. The brochures used a “traffic light” color coding scheme with the green brochure indicating readiness to be screened for CRC (preparation for action stage), the yellow brochure indicating considering becoming screened (contemplation stage), and the red brochure indicating not considering screening (pre-contemplation stage). The brochure also included sigmoidoscopy in addition to FOBT and colonoscopy as sigmoidoscopy was another commonly used CRC screening test in the clinic. After a brochure was selected, patients were encouraged to show the brochure to their physician and to discuss their preferences and readiness for screening.

### Data collection

The pre-survey lasted about 10 min and was administered through phone in English or Spanish. When both the pre-survey of the patients and their alters were completed, the community health worker met with the patients at the clinic within 4 weeks of completing the survey to view the CRC decision aid and to share the accompanying brochures. The patients navigated the video on their own and communicated with the community health workers using the selected color brochure. The community health worker was instructed to encourage the participants to show the brochure to their physician and to discuss their preferences and readiness for screening. Two months after viewing the decision aid, the community health worker called the patients to administer the post survey including whether they had talked to their physicians about CRC screening, received CRC screening, or talked to their alters about CRC screening.

The pre-survey for alters were also administered on the phone in English or Spanish. The community health workers administered the post-survey via phone to ask whether the alter received the cancer communication from the patient who referred them to participate in the study; the alter’s post-survey was conducted after the completion of the patients’ post-survey. Both the pre- and the post-survey data collection lasted about 10 min.

### Measures

There were three outcome variables. The primary outcome was influence of the decision aid on *desire to screen for CRC* among patients. This variable was assessed at post-survey by asking patients one question: “Did the information from the video influence your desire to get screened for CRC?” Response options included “yes,” “no,” and “don’t know/not sure.”

The second outcome was *intention to screen for CRC* among alters. This variable was assessed by asking the alters their plans on getting a CRC screening using either FOBT/FIT, colonoscopy, or sigmoidoscopy. The question included four response options, following previous studies [9, 26]: “definitely planning to be screened,” “thinking about getting screened but not planning,” “not thought about getting screened” and “don’t know/not sure.” The responses were dichotomized into “planning to be screened” and “not planning to be screened.” Not planning to be screened included “thinking about getting screened,” “not thought about getting screened,” and “don’t know/not sure due to the small sample size.”

The third outcome was patients’ *communication with their alters* (i.e., family members, friends, or co-workers) about CRC screening. This variable was captured with two questions. Prior to asking the questions, the community health worker stated the first name of the alter who was participating in the research study to the patient and asked to keep *this person* in mind when answering the questions. The first question asked, “Since watching the video, have you shared information about cancer screening with this person?” with a ‘yes’ or ‘no’ response option. If the patient answered, ‘yes’ to the first question, then the community health worker asked the second question: “If so, for which type of cancer?” When the patient had more than one alter, these questions were asked separately for each alter.

Demographics included age, race/ethnicity, sex (male or female), marital status (the five response options were collapsed into married and unmarried), education (the six response options were collapsed into eighth grade or less, some high school or general education development (GED), and some college or more), employment (the six response categories were collapsed into full-time, part-time, and unemployed/homemaker/retired/disability), country of birth, years in the U.S., and health insurance (the five response options were collapsed into yes and no). General health status was captured with five response options, which were collapsed into excellent/very good/good and fair/poor.

Knowledge about CRC was assessed based on participants’ ability to correctly recall the information from the decision aid including 1) age at which one should begin CRC screening [correct answer: 50 (based on the Healthy People 2020 at the time of survey], 2) number of CRC screening methods discussed in the decision aid [correct answer: more than one method], and 3) lifetime risk for CRC [correct answer: less than 10 percent].

Information processing was captured by measuring information recall and information influence. Information recall was assessed by asking the participants whether they remembered the contents of the CRC decision aid; response options were “yes” or “no.” Information influence was measured by asking whether the participants thought the video influenced their desire to get screened for CRC; response options were “yes” or “no.”

Social support was assessed with five questions: 1) Who do you feel close to? 2) During a minor everyday upset, who do you turn to for support? 3) When you want to talk about your feelings, who are you comfortable talking with? 4) In general, whose advice do you take? 5) Is there someone whose suggestions you feel you must follow? All five questions included single-option responses: spouse, relative, children, close friend, co-worker, and others. We also asked how participants shared information with their alters, with single-option responses including face-to-face, Email, phone, social media (e.g., Twitter, Facebook, Blog), and others.

### Data analysis

We calculated descriptive statistics (mean and percentages) from the pre-survey to characterize participants and their alters. We used a Fisher’s exact test to examine the relationship between information recall, knowledge, and information influence and participants’ intention to receive a CRC screening test. We also conducted a Fisher’s exact test to examine the relationship between information shared by participants and alters’ CRC screening intention. Analysis was performed using STATA version 15.0. with an alpha level of 0.05.

## Results

### Participant and alter characteristics

Patients’ mean age was 61 years (SD: 4.7 years) (Table 1). About half of the patients were male (54.8%) and more than half were married (59.5%). Most had an eighth-grade level of education or less (71.4%) and about half of the patients were employed (52.4%). The majority reported being born outside the U.S. (71.4%) and having lived in the U.S. for about 35 years on average (SD: 8.4 years). The majority of patients reported having health insurance (90.5%), and one third (33.3%) reported their health as being good or better. Most patients reported feeling close to family and close friends and that they turn to them for support when they are upset, need to talk about their feelings, or need advice, and they follow their advice.

**Table 1:** Patients’ and alters’ demographics, health, and social support characteristics

	**Patients** **(*n* = 42)**	**Alters** **(*n* = 19)**^**e**^
**Age (Mean, SD)**	60.7 (4.7)	56.2 (7.4)
**Hispanic/Latino**		
Yes	42 (100.0%)	15 (78.9%)
No	0	4 (21.1%)
**Sex**		
Male	23 (54.8%)	7 (36.8%)
Female	19 (45.2%)	12 (63.2%)
**Marital status**		
Married	25 (59.5%)	15 (78.9%)
Unmarried	17 (40.5%)	4 (21.1%)
**Education**		
Eighth grade or less	30 (71.4%)	11 (57.9%)
Some high school or GED	5 (11.9%)	5 (26.3%)
Some college or more	7 (16.7%)	3 (15.8%)
**Employment**		
Full-time	18 (42.9%)	9 (47.4%)
Part-time	4 (9.5%)	3 (15.8%)
Unemployed/homemaker/retired/disability	20 (47.6%)	7 (15.8%)
**Country of birth**		
U.S	12 (28.6%)	5 (26.3%)
Mexico	30 (71.4%)	12 (63.2%)
El Salvador	0	2 (10.5%)
**Years in the U.S.** (Mean, SD)	34.6 (8.4)	30.4 (10.8)
**Health insurance**		
Yes	38 (90.5%)	16 (84.2%)
No	4 (9.5%)	3 (15.8%)
**General health status**		
Excellent/very good/good	14 (33.3%)	10 (52.6%)
Fair/poor	28 (66.7%)	9 (47.4%)
**Social support**		
*“Who do you feel close to?”*		
Spouse	24 (57.1%)	10 (52.6%)
Relative	12 (28.6%)	2 (10.5%)
Children	1 (2.4%)	4 (21.1%)
Close friend	3 (7.1%)	2 (10.5%)
Other^a^	2 (4.8%)	1 (5.3%)
*“During a minor everyday upset, who do you turn to for support?”*		
Spouse	19 (45.2%)	12 (63.2%)
Relative	13 (30.9%)	3 (15.8%)
Children	2 (4.8%)	2 (10.6%)
Close friend	3 (7.1%)	1 (5.3%)
Other^b^	4 (9.5%)	1 (5.3%)
*“When you want to talk about your feelings, who are you comfortable talking with?”*		
Spouse	22 (52.4%)	7 (36.8%)
Relative	11 (26.2%)	2 (10.5%)
Children	2 (4.8%)	2 (10.5%)
Close friend	6 (14.3%)	4 (21.1%)
Other^c^	1 (2.4%)	4 (21.1%)
*“In general, whose advice do you take?”*		
Spouse	16 (38.1%)	7 (36.8%)
Relative	12 (28.6%)	5 (26.3%)
Children	0	2 (10.5%)
Close friend	5 (11.9%)	3 (15.8%)
Co-worker	3 (7.1%)	0
Other^d^	4 (9.5%)	2 (10.5%)
* “Is there someone whose suggestions you feel you must follow?”*		
Spouse	15 (35.7%)	6 (31.6%)
Relative	15 (35.7%)	4 (21.1%)
Close friend	4 (9.5%)	5 (26.3%)
Co-worker	3 (7.1%)	0
Other	5 (11.9%)	0

There were two missing values for age

^a^Patients = co-worker; alters = siblings

^b^Patients = self; alters = alcoholics anonymous group

^c^Patients = co-worker; alters = siblings and self

^d^Patients = family, relative, friend, and co-worker; alters = family and relative

^e^10 participants had alters with 1–3 alters per participant

Alters’ mean age was 56 years (SD: 7.4 years). Many were Hispanic (78.9%), female (63.2%), married (78.9%), had an eighth-grade level education or less (57.9%), and were employed on a full- or part-time basis (63.2%). Many reported being born outside the U.S. (73.7%) and having lived in the U.S. for 30 years on average (SD: 10.8 years). Most reported having health insurance (84.2%) and about half reported their health as being good or better (52.6%). Most alters reported feeling close to their spouse and receiving spousal support when upset, but when asked about communicating feelings, taking advice, and following suggestions, alters also mentioned relatives and close friends.

### Influence of decision aid on patients’ desire to screen for CRC

All but one of the patients reported remembering the content of the decision aid (97.6%); however, many could not recall specific health information covered in the decision aid. Most (88.1%) recalled the correct information about the number of CRC screening methods discussed in the decision aid; about two thirds (66.7%) reported correct information about the recommended age for receiving the first CRC screening test and only 14.3% reported correct information on the lifetime risk of having a CRC.

Among the 41 patients who answered correctly to the knowledge question, the majority (95.1%) reported that, in general, the video influenced their own desire to screen for CRC. Table 2 (which includes analysis of the post-survey data) illustrates the relationship between patients’ acquired knowledge about CRC through the decision aid and influence of the decision aid on their desire to screen for CRC. Although not statistically significant, patients who correctly identified the number of CRC screening methods and the lifetime risk of developing CRC were more likely to report that the decision aid influenced their desire to screen for CRC.

**Table 2:** Patients’ knowledge about CRC acquired by the decision aid on their desire to screen for CRC (*n* = 41)

	**Desire to screen for CRC**		**Total**
	No	Yes	
**Knowledge**			
*Age of CRC screening (p* = *0.323)*			
Incorrect	0	13 (100.0%)	13
Correct	2 (7.1%)	26 (92.9%)	28
Total	2	39	41
*Number of CRC screening method (p* = *0.188)*			
Incorrect	1 (25.0%)	3 (75.0%)	4
Correct	1 (2.7%)	36 (97.3%)	37
Total	2	39	41
*Risk of getting CRC in lifetime (p* = *0.548)*			
Incorrect	2 (5.7%)	33 (94.3%)	35
Correct	0	6 (100.0%)	6
Total	2	39	41

Fisher’s exact tests were applied. Missing data (*n* = 1) was not included in the analysis

Post-surveys were used

*CRC* Colorectal cancer screening

### The communication about decision aids and alters’ CRC screening intention

Most patients reported sharing information about CRC with their alters after viewing the decision aid (88.9%). Face-to-face was the most common way of communicating about CRC (66.7%), followed by phone (44.4%).

Conversely, among 19 alters, sixty-eight percent (*n* = 13) reported that they received CRC information from the patients. Alters reported that information was shared mostly via face-to-face interactions (92.3%) and a few mentioned sharing information via phone (15.4%). Table 3 presents the relationship between patients’ report of CRC communication and alters’ intention to be screened for CRC. Patients’ report of CRC communication from the decision aid to their alters was significantly related to alters’ intention to get screened for CRC using FOBT and sigmoidoscopy. Alters of patients who reported sharing the CRC screening information showed a higher intention to undergo FOBT compared to alters of patients who reported not sharing information about CRC screening (90% vs 0%; *p* = 0.014). Similarly, strong evidence was also found for sigmoidoscopy (66.7% vs. 0%; *p* = 0.011). For colonoscopy, while patient-alter CRC communication led to increased report of intention to screen for colonoscopy among alters, compared to those who did not report patient-alter CRC communication (87.5% vs. 50.0%), it was not statistically significant (*p* = 0.378).

**Table 3:** The relationship between patients’ CRC screening communication on alters’ intention to be screened for CRC (*n* = 19)

	**Shared screening information**		**Total**
	No	Yes	
*Plan to get FOBT*^a^			
Not planning	3 (100.0%)	1 (10.0%)	4
Planning	0	9 (90.0%)	9
Total	3	10	13
*Plan to get colonoscopy*^b^			
Not planning	1 (50.0%)	1 (12.5%)	2
Planning	1 (50.0%)	7 (87.5%)	8
Total	2	8	10
*Plan to get sigmoidoscopy*^c^			
Not planning	6 (100.0%)	4 (33.3%)	10
Planning	0	8 (66.7%)	8
Total	6	12	18

Fisher’s exact tests were applied

Patients’ and alters’ post-surveys were used

^a^*n* = 13, *p* = *0.014*

^b^*n* = 10, *p* = *0.378*

^c^*n* = 18, *p* = *0.011*

## Discussion

This study evaluated the effect of a patient decision aid on Hispanic patients’ CRC screening intention and their communication about CRC with their alters. In addition, it examined the association between patient-alter communication about CRC on their alters’ intention to get screened for CRC. While preliminary in nature, this is one of the first studies to demonstrate dissemination of cancer information within social networks capturing the perspectives of patient and their social network.

Our study provides proof-of-concept that there is an exchange of CRC communication between patients and their alters with a preference for face-to-face communication, and patient-alter CRC communication leads to intention to screen for CRC screening among social networks. These findings are corroborated by results from previous studies that show interpersonal communication around cancer screening is common and, in some cases, can lead to cancer screening [27–30]. Our study also shows that patients preferred to discuss CRC screening via face-to-face communication with their social network instead of communicating using social media (such as Twitter, Facebook, and blogs). A study that examined willingness to use social media and emails for peer-to-peer cancer screening communication among 438 insured individuals from three large health plans found that across all age groups (40–73 years old), patients were more willing to share CRC screening experiences through conversations (74%) than emails (41%) or other e-communication (i.e., texting, Facebook, instant messaging, Internet-based chat, and video chatting) (25%) [31].

While the use of social media has exponentially increased as a tool to connect with others and sharing people’s life experiences [32], this may not extend to sharing personal health information. Cutrona and colleagues [31] note that sharing information about health through social media may be dependent on age. The study found a slightly higher willingness to use e-communication to share personal CRC information among the younger participants (28% and 29% among 40–49 and 50–59 years old, respectively) compared to older participants (19.6% among 60–73 years old). If this life course pattern continues, the comfort around sharing personal CRC information may shift from face-to-face conversation to e-communication. The average age of our study participants was 62 years old; they were also all Hispanics with the majority primarily speaking Spanish (70%). Studies have extensively reported on Hispanics’ cultural value for personalism, that is, the preference for person-to-person interaction [33, 34]. Future studies should examine whether cultural values such as personalism trumps over the changing communication landscape around health communication among Hispanics.

Our findings also suggest that patients’ exposure to the decision aid was associated with their own CRC screening decisions, as well as with alters’ intention to be screened for CRC. The impact of patient decision aids on patient’s own CRC knowledge [35] and screening behavior has been previously reported [10, 13, 36]. What has been less studied (but is examined in this study) was the effect of the decision aid on alters’ CRC screening decision. It is important to note that patients who reported communicating with their alters about CRC screening did not always correctly recall the information about CRC screening. Regardless of the patients’ ability to correctly recall the information, our study suggests there is value in the exchange of CRC communication between patients and their alters, as it may lead to changes in CRC screening decisions to their social network. Future studies should examine the content of this conversation, including what is being communicated during these interactions, the exchange of “correct” versus “incorrect” information, and whether specific information impacts alters’ intention to be screened (vs. general communication) and, ultimately, their CRC screening decision. Additionally, given the new recommendations of the United States Preventive Services Task Force, lowering CRC screening starting age from 50 to 45 among those at average risk for CRC [37, 38], and studies demonstrating screening benefits among adults aged 45 to 49 years [39, 40], future research may explore CRC screening communication within social networks among younger age population and whether communication mode differs among older and younger population.

To our knowledge, this is the first study to evaluate a CRC screening patient decision aid beyond the individual receiving it. There are several limitations to this study. First, we had a small sample size of participants and alters. Second, our study evaluated the decision aid using one-arm pre/post design instead of a randomized controlled trial. Third, the recruitment strategy used for alters, requesting them to contact the study team yielded a low participation rate. Designing an active recruitment approach where the research team gathers the alter’s contact information from the patients and initiating the contact with the alter may lead to improved recruitment in the future. Fourth, while the majority of patients (95.1%) responded that the video was influential in their desire to screen for CRC, we did not directly ask whether the influence was positive or negative. Finally, all data are based on self-report, including the CRC screening questions. Validation with electronic medical records is warranted in future studies.

## Conclusion

Patient decision aids have the potential to be used as an effective tool to engage Hispanic patients and their alters to share health information. Interpersonal communication through face-to-face interactions could be a medium for CRC screening communication among Hispanic patients.

A larger future study is needed using a randomized controlled design to evaluate the effectiveness of the knowledge gained from the use of patient decision aids on CRC screening behavior beyond the recipient of the decision aid to their social network.

## Supplementary Information

Supplementary Material 1: Appendix A: Pre-survey questionnaire.

Supplementary Material 2: Appendix B: The intervention brochure.

## Data Availability

The datasets used and/or analyzed during the current study are available from the corresponding author on reasonable request.
