# Supplementary material for: Dissemination of colorectal cancer information among Hispanic patients and their social network
Source: BMC Public Health. 2024 Sep 27;24:2616. doi: 10.1186/s12889-024-20095-7 (PMC11437630; doi:10.1186/s12889-024-20095-7)
Supplement: Supplementary file 1 — Supplementary Material 1: Appendix A: Pre-survey questionnaire. [file 12889_2024_20095_MOESM1_ESM.pdf]

FRED HUTCHINSON CANCER RESEARCH CENTER
A LIFE OF SCIENCE

Participant ID #: ________________

Date: _____/ _____/_____

Interviewer: ___________________

PROJECT CHAT
PATIENT BASELINE SURVEY

HEALTH QUESTIONS

H1. In general, would you say your health is excellent, very good, good, fair, or poor?

- ☐1 Excellent
- ☐2 Very good
- ☐3 Good
- ☐4 Fair
- ☐5 Poor
- ☐888 DON'T KNOW / NOT SURE
- ☐999 REFUSED

COLON HEALTH

CH1. Next, I'd like to talk about cancer of the colon. The colon is the same thing as your large intestine. Have you ever heard of colon cancer, that is, cancer of the large intestine?

- ☐1 Yes
- ☐2 No
- ☐888 DON'T KNOW / NOT SURE
- ☐999 REFUSED

CH2. Have you ever heard of a colon polyp?

- ☐1 Yes
- ☐2 No
- ☐888 DON'T KNOW / NOT SURE
- ☐999 REFUSED

CH3. Please try to respond to the following question to the best of your ability. Out of 100 adults, how many will have colon cancer in their lifetime?

- ☐1 Less than 10
- ☐2 10 to 19
- ☐3 20 to 29
- ☐4 30 or more
- ☐888 DON'T KNOW / NOT SURE
- ☐999 REFUSED

CH4. At what age is it recommended that a person should start getting screened for colon cancer?

- ☐1 35
- ☐2 40
- ☐3 45
- ☐4 50
- ☐5 55
- ☐888 DON'T KNOW / NOT SURE
- ☐999 REFUSED

Participant ID #: ________________

Date: _____/ _____/_____

Interviewer: ___________________

CH5. There is only one test for the early detection of colon cancer. Do you agree, disagree, or are you not sure?

- ☐1 Agree
- ☐2 Disagree
- ☐3 Not sure
- ☐888 DON'T KNOW / NOT SURE
- ☐999 REFUSED

FECAL OCULT BLOOD TEST (FOBT)

Now, I would like to ask you questions about some medical tests. There are 3 tests recommended for colon cancer screening. It is generally recommended that you get one of these tests. The first test is called a fecal occult blood test also known as a stool blood test. It is done at home using a set of 3 cards to determine whether the stool contains blood. The following questions are about this test. For each question, please choose the answer choice that is best for you.

FE1. Do you plan to talk to your doctor or someone in the clinic about getting a fecal occult blood test?

- ☐1 I am definitely planning to talk to my doctor about the FOBT
- ☐2 I am thinking about talking with my doctor about the FOBT
- ☐3 I have not thought about talking to my doctor about the FOBT
- ☐888 DON'T KNOW / NOT SURE
- ☐999 REFUSED

FE2. Do you plan to get a fecal occult blood test in the next 12 months?

- ☐1 I am definitely planning to have an FOBT
- ☐2 I am thinking about having an FOBT
- ☐3 I have not thought about having an FOBT
- ☐888 DON'T KNOW / NOT SURE
- ☐999 REFUSED

Now I'd like to ask your opinions about the fecal occult blood test. We're interested in what you think. There are no right or wrong answers. I'll read some statements and for each, please tell me whether you agree, disagree, or don't know.

	Agree	Disagree	DK/NS	REFUSED
**FE3.** It's not possible to do a colon cancer screening test at home.	☐1	☐2	☐888	☐999
**FE4.** The fecal occult blood test, or FOBT, should be done every three years.	☐1	☐2	☐888	☐999
**FE5.** A fecal occult blood test for finding colon cancer is not very effective.	☐1	☐2	☐888	☐999
**FE6.** A fecal occult blood test takes too much time.	☐1	☐2	☐888	☐999

Participant ID #: ________________

Date: _____/ _____/_____

Interviewer: ___________________

COLONOSCOPY

The next set of questions is about colonoscopy, another test to check for colon cancer. This test examines the entire colon using a narrow, lighted tube that is inserted in the rectum. For each of the following questions, please choose the answer choice that is best for you.

CO1. Are you planning on talking to your doctor or someone at the clinic about getting a colonoscopy?

- ☐1 I am definitely planning on talking to my doctor about the test
- ☐2 I am thinking about talking to my doctor about the test
- ☐3 I have not thought about talking to my doctor about the test
- ☐888 DON'T KNOW / NOT SURE
- ☐999 REFUSED

CO2. Are you planning on getting a colonoscopy?

- ☐1 I am definitely planning on getting a colonoscopy
- ☐2 I am thinking about getting a colonoscopy
- ☐3 I have not thought about getting a colonoscopy
- ☐888 DON'T KNOW / NOT SURE
- ☐999 REFUSED

Now, I'd like your opinions about colonoscopy. Again, we're interested in what you think, and there are no right or wrong answers. For each statement I read, please tell me whether you agree, disagree, or don't know.

	Agree	Disagree	DK/NS	REFUSED
**CO3.** A person can drive their car to go home or to work immediately after a colonoscopy.	☐1	☐2	☐888	☐999
**CO4.** There is no risk during the colonoscopy process.	☐1	☐2	☐888	☐999
**CO5.** The preparation for a colonoscopy is easy.	☐1	☐2	☐888	☐999
**CO6.** The colonoscopy test for finding colon cancer is not very effective.	☐1	☐2	☐888	☐999
**CO7.** Having a colonoscopy test is very embarrassing.	☐1	☐2	☐888	☐999

SIGMOIDOSCOPY

The next set of questions is about sigmoidoscopy, also another test to check for colon cancer. It also uses a lighted tube, similar to the colonoscopy, but it only examines the left side of the colon and not the entire colon. Again, for each of the following questions, please choose the answer choice that is best for you.

SG1. Are you planning on talking to your doctor or someone at the clinic about getting a sigmoidoscopy?

- ☐1 I am definitely planning on talking to my doctor about the test
- ☐2 I am thinking about talking to my doctor about the test
- ☐3 I have not thought about talking to my doctor about the test
- ☐888 DON'T KNOW / NOT SURE
- ☐999 REFUSED

SG2. Are you planning on getting a sigmoidoscopy?

- ☐1 I am definitely planning on getting a sigmoidoscopy
- ☐2 I am thinking about getting a sigmoidocopy
- ☐3 I have not thought about getting a sigmoidoscopy
- ☐888 DON'T KNOW / NOT SURE
- ☐999 REFUSED

EXPOSURE TO MEDIA

M1. In the last 6 months, have you seen any brochures, newspaper articles, radio programs, or TV Ads on colon cancer screening?

- ☐1 Yes
- ☐2 No
- ☐888 DON'T KNOW / NOT SURE
- ☐999 REFUSED

M2. Do you have a DVD player, VCR, computer, or internet access in your home? Tell me all the ones you have.

- ☐1 DVD
- ☐2 VCR
- ☐3 Computer
- ☐4 Internet access
- ☐888 DON'T KNOW / NOT SURE
- ☐999 REFUSED

COMPUTER USE

C1. In general, how comfortable do you feel using the computer?

- ☐1 Very comfortable
- ☐2 A little comfortable
- ☐3 A little uncomfortable
- ☐4 Very uncomfortable
- ☐5 Don't know how to use the computer
- ☐888 DON'T KNOW / NOT SURE
- ☐999 REFUSED

SOCIAL NETWORK CHARACTERISTICS

Now, we want to ask you some questions about people who you may socialize with including family members, relatives, friends, and co-workers.

SN1. Have you ever shared information about cancer screening with any of your family members, relatives, friends, or co-workers?

- ☐1 Yes
- ☐2 No [**SKIP TO SN5**]
- ☐888 DON'T KNOW / NOT SURE [**SKIP TO SN5**]
- ☐999 REFUSED [**SKIP TO SN5**]

SN1a. If so, which type of cancer? ______________________________

SN2. Who did you share the information about cancer screening? **[CHECK ALL THAT APPLY]**

- ☐1 Family member
- ☐2 Relative
- ☐3 Friend
- ☐4 Co-worker
- ☐5 Other, specify (**SN2e**): ____________________________
- ☐888 DON'T KNOW / NOT SURE
- ☐999 REFUSED

SN3. When did you share the information about cancer screening?

- ☐1 A week ago or less
- ☐2 More than a week ago, but not more than 1 month ago
- ☐3 More than 1 but not more than 2 months ago
- ☐4 More than 2 but not more than 6 months ago
- ☐5 More than 6 months ago but not more than 1 year ago
- ☐6 More than 1 year ago
- ☐888 DON'T KNOW/ NOT SURE
- ☐999 REFUSED

SN4. How did you share this information? Through face-to-face conversation, email, phone, or post on social media such as Twitter, Facebook, and blog? **[CHECK ALL THAT APPLY]**

- ☐1 Face-to-face
- ☐2 Email
- ☐3 Phone
- ☐4 Social Media (Twitter, Facebook, blog)
- ☐5 Other, specify (**SN4e**):____________________________
- ☐888 DON'T KNOW/ NOT SURE
- ☐999 REFUSED

Continue to next page

Participant ID #: ________________

Date: _____/ _____/_____

Interviewer: ___________________

For the next set of questions, please tell me about people around you whether is your spouse, relative, close friend, co-worker, acquaintance, or others. **[IF PARTICIPANT ANSWERS "OTHER", ASK THEM TO SPECIFY]**

	Spouse	Relative	Close Friend	Co-worker	Acquaintance	Other, specify:	DON' T KNOW	REFUSED
**SN5.** During a minor everyday upset, who do you turn to for support?	☐1	☐2	☐3	☐4	☐5	☐6 ______	☐888	☐999
**SN6.** Who do you feel close to?	☐1	☐2	☐3	☐4	☐5	☐6 ______	☐888	☐999
**SN7.** When you want to talk about your feelings, who are you comfortable talking with?	☐1	☐2	☐3	☐4	☐5	☐6 ______	☐888	☐999
**SN8.** In general, whose advice do you take?	☐1	☐2	☐3	☐4	☐5	☐6 ______	☐888	☐999
**SN9.** Is there someone whose suggestions you feel you must follow?	☐1	☐2	☐3	☐4	☐5	☐6 ______	☐888	☐999

SN10. You identified two people who you socialize with to participate in this research study with you. Now, I will ask you questions about ___________________ **[FIRST NAME]**. Please keep this person in mind when answering the following questions.

How long have you known this person? __________year(s) __________month(s)

SN11. Is this person, a spouse, relative, close friend, co-worker, or other?

☐1 Spouse
☐2 Relative
☐3 Close friend
☐4 Co-worker
☐5 Acquaintance
☐6 Other, specify (**SN11f**): _______________________
☐888 DON'T KNOW / NOT SURE
☐999 REFUSED

Continue to next page

Participant ID #: ________________

Date: _____/ _____/_____

Interviewer: ___________________

SN12. In general, how often do you talk to this person?

- ☐1 Never
- ☐2 Rarely
- ☐3 Sometimes
- ☐4 Often
- ☐5 Very often
- ☐888 DON'T KNOW / NOT SURE
- ☐999 REFUSED

SN13. How often do you talk to this person about health related topics or issues?

- ☐1 Never
- ☐2 Rarely
- ☐3 Sometimes
- ☐4 Often
- ☐5 Very often
- ☐888 DON'T KNOW/ NOT SURE
- ☐999 REFUSED

SN14. Have you ever shared with this person information about cancer screening?

- ☐1 Yes
- ☐2 No [**SKIP TO SN17**]
- ☐888 DON'T KNOW/ NOT SURE [**SKIP TO SN17**]
- ☐999 REFUSED [**SKIP TO SN17**]

SN14a. If so, which type of cancer? _______________

SN15. When did you share the information about cancer screening?

- ☐1 A week ago or less
- ☐2 More than a week ago, but not more than 1 month ago
- ☐3 More than 1 but not more than 2 months ago
- ☐4 More than 2 but not more than 6 months ago
- ☐5 More than 6 months ago but not more than 1 year ago
- ☐6 More than 1 year ago
- ☐888 DON'T KNOW/ NOT SURE
- ☐999 REFUSED

SN16. How did you share this information? Through face-to-face conversation, email, phone, or post on social media such as Twitter, Facebook, and blog? **[CHECK ALL THAT APPLY]**

- ☐1 Face-to-face
- ☐2 Email
- ☐3 Phone
- ☐4 Social Media (Twitter, Facebook, blog)
- ☐5 Other, specify (**SN16e**): ____________________________
- ☐888 DON'T KNOW / NOT SURE
- ☐999 REFUSED

Participant ID #: ________________

Date: _____/ _____/_____

Interviewer: ___________________

SN17. Now, I will ask you to think about the other person ______________ **[FIRST NAME]**. Please keep this person in mind when answering the following questions.

How long have you known this person? ___________year(s) ___________month(s)

SN18. Is this person, a spouse, relative, close friend, co-worker, or other?

- ☐1 Spouse
- ☐2 Relative
- ☐3 Close friend
- ☐4 Co-worker
- ☐5 Acquaintance
- ☐6 Other, specify (**SN18f**): __________________________
- ☐888 DON'T KNOW / NOT SURE
- ☐999 REFUSED

SN19. How often do you talk to this person in general?

- ☐1 Never
- ☐2 Rarely
- ☐3 Sometimes
- ☐4 Often
- ☐5 Very often
- ☐888 DON'T KNOW / NOT SURE
- ☐999 REFUSED

SN20. How often do you talk to this person about health related topics or issues?

- ☐1 Never
- ☐2 Rarely
- ☐3 Sometimes
- ☐4 Often
- ☐5 Very often
- ☐888 DON'T KNOW / NOT SURE
- ☐999 REFUSED

SN21. Have you ever shared with this person information about cancer screening?

- ☐1 Yes
- ☐2 No
- ☐888 DON'T KNOW / NOT SURE
- ☐999 REFUSED

SN21a. If so, which type of cancer? _______________

Continue to next page

Participant ID #: ______________

Date: _____/ _____/_____

Interviewer: _________________

SN22. How did you share this information? Through face-to-face conversation, email, phone, or post on social media such as Twitter, Facebook, and blog? **[CHECK ALL THAT APPLY]**

- ☐1 Face-to-face
- ☐2 Email
- ☐3 Phone
- ☐4 Social Media (Twitter, Facebook, blog)
- ☐5 Other, specify (**SN22e**): ________________________
- ☐888 DON'T KNOW / NOT SURE
- ☐999 REFUSED

SOCIODEMOGRAPHIC FACTORS

SD1. In what country where you born? ________________ **[IF BORN IN THE USA, SKIP TO SD4]**

SD2. How old were you when you came to the US?

- ☐1 Age _________ (0-99 = Age)
- ☐888 DON'T KNOW / NOT SURE
- ☐999 REFUSED

SD3. In total, how many years have you lived in the US?

- ☐1 Total years ________ (0-99 = Total years)
- ☐888 DON'T KNOW / NOT SURE
- ☐999 REFUSED

SD4. How many years of school did you finish?

- ☐1 Never went to school or just Kindergarten
- ☐2 Grades 1 through 8
- ☐3 Grades 9 through 11
- ☐4 Grade 12 or GED (High School Diploma Certificate)
- ☐5 College 1 to 3 years
- ☐6 College 4 or more years
- ☐888 DON'T KNOW / NOT SURE
- ☐999 REFUSED

SD5. Do you have a way to cover your medical costs, such as medical insurance, HMOs, or government plans such as Medicare and Medicaid?

- ☐1 Yes
- ☐2 No [**SKIP TO SD7**]
- ☐888 DON'T KNOW / NOT SURE [**SKIP TO SD7**]
- ☐999 REFUSED [**SKIP TO SD7**]

Continue to next page

Participant ID #: ________________

Date: _____/ _____/_____

Interviewer: ___________________

SD6. What kind of medical insurance do you have?

- ☐1 Private Insurance such as Medcost, United Healthcare, or Blue Cross/Blue Shield
- ☐2 Medicare
- ☐3 Medicaid
- ☐4 Medicare AND Medicaid
- ☐5 Other, specify (**SD6e**): ________________________
- ☐888 DON'T KNOW / NOT SURE
- ☐999 REFUSED

SD7. Are you working full time, working part time, unemployed, a homemaker, retired, or unable to work due to poor health or disability?

- ☐1 Working full time
- ☐2 Working part time
- ☐3 Unemployed
- ☐4 Homemaker or Caretaker
- ☐5 Retired
- ☐6 Unable to work due to poor health or disability
- ☐888 DON'T KNOW / NOT SURE
- ☐999 REFUSED

SD8. Are you currently married or living with a partner, separated, divorced, widowed, or never married?

- ☐1 Married
- ☐2 Divorced
- ☐3 Widowed
- ☐4 Separated
- ☐5 Single, never married
- ☐6 Not married but living with a partner
- ☐888 DON'T KNOW / NOT SURE
- ☐999 REFUSED

SD9. Now I am going to ask you about your household income. It may be hard to estimate this income, but do your best. This information will be strictly confidential. Taking all the income of members of your household, which of these categories best fits your total household income for last year (2013)? Please stop me when I get to the right category. Is it…

- ☐1 Less than $10,000
- ☐2 $10,000 up to $19,999
- ☐3 $20,000 up to $29,999
- ☐4 $30,000 up to $49,999
- ☐5 $50,000 up to $69,999
- ☐6 $70,000 up to $99,999
- ☐7 $100,000 up to $149,999
- ☐8 $150,000 or more
- ☐888 DON'T KNOW / NOT SURE
- ☐999 REFUSED

SD10. What is your date of birth? Month______/Day_______/Year__________

Continue to next page

Participant ID #: ________________

Date: _____/ _____/_____

Interviewer: ___________________

SD11. Are you a:

- ☐1 Man
- ☐2 Woman
- ☐888 DON'T KNOW / NOT SURE
- ☐999 REFUSED

SD12. Do you consider yourself to be of Latino or Hispanic origin?

- ☐1 Yes
- ☐2 No
- ☐888 DON'T KNOW / NOT SURE
- ☐999 REFUSED

SD13. What ethnic group or race do you belong to (select the best answer for you)?

- ☐1 White/Caucasian
- ☐2 Black/African-American
- ☐3 American-Indian/Alaska Native
- ☐4 Asian/Pacific Islander
- ☐5 Other, specify (**SD13e**): ____________________
- ☐888 DON'T KNOW / NOT SURE
- ☐999 REFUSED

[READ]: Thank you, we are finished with the survey!
