# Supplementary material for: Dissemination of colorectal cancer information among Hispanic patients and their social network
Source: BMC Public Health. 2024 Sep 27;24:2616. doi: 10.1186/s12889-024-20095-7 (PMC11437630; doi:10.1186/s12889-024-20095-7)

We’re glad to hear that you want to get a colon cancer screening test

	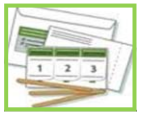 **FOBT**	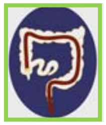 **Colonoscopy**	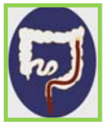 **Sigmoidoscopy**
**Effectiveness**	Medium	High	High
**Cost**	$5 - 60*<br>**Available at no cost at certain clinics*	$3,500 - $4,700*<br>**Covered by most insurance plans. Patient responsible for copay or deductible*	$187 - $246*<br>**Covered by most insurance plans. Patient responsible for copay or deductible*
**Time**	10 - 20 minutes	1½ days	½ day
**Frequency**	Every year	Every 10 years	Every 5 years
**Discomfort**	Low	Medium	Medium
**Complication risk**	0	1 in 1000	1 in 7500

SHOW THIS BROCHURE TO YOUR DOCTOR

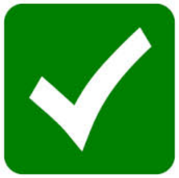 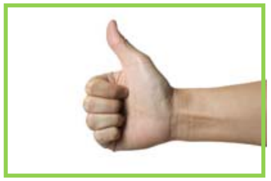

I'm ready to get a test.

My preference is:

☐ **FOBT**

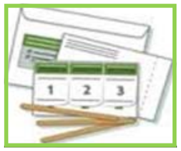

☐ **Colonoscopy**

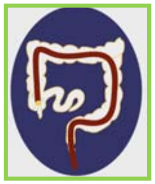

☐ **Sigmoidoscopy**

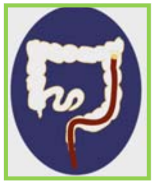

☐ **Don't have a preference, I just want to get a test.**

Show this brochure to your doctor

Show this brochure to your doctor

Dear medical provider:

Your patient recently viewed a video regarding colon cancer screening. By choosing this brochure, your patient has indicated that he/she is ready to be screened for colon cancer and has chosen a preferred testing method marked in the front of this brochure. The inside of this brochure contains a comparison table that may be useful as your patient proceeds to be screened for colon cancer.

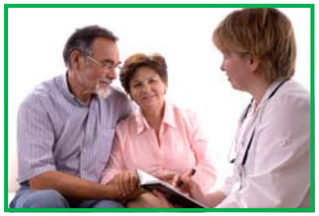

PROJECT CHAT

Linda K. Ko, PhD
Center for Health Communication Intervention (CHealCI)
Fred Hutchinson Cancer Research Center
1100 Fairview Ave. N
Seattle, WA 98019

Phone: **1-855-682-7998**

Email: **earodrig@fhcrc.org**

Web: **http://research.fhcrc.org/chealci/en.html**

Colon cancer screening tests

The **FOBT**, or stool test, is done at home using special cards to determine if the stool sample contains blood.

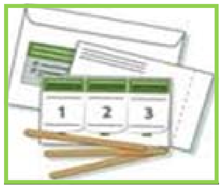

In the **colonoscopy** test, a narrow and illuminated tube is inserted through the rectum and is used to examine <u>the entire colon</u>. It's necessary to take a day off from work and someone has to drive you home after the test.

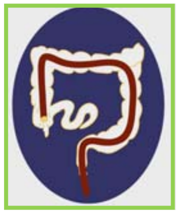

In the **sigmoidoscopy** test, a narrow and illuminated tube is inserted through the rectum and is used to examine <u>the lower portion colon</u>. It's necessary to take the morning off from work and someone has to drive you home after the test.

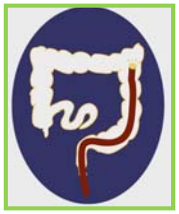

When you get a colon cancer screening test, you will feel that you made the right choice for you and your family

	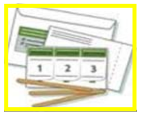 FOBT	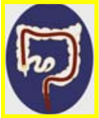 Colonoscopy	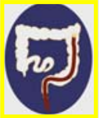 Sigmoidoscopy
**Effectiveness**	Medium	High	High
**Cost**	$5 - 60*<br>**Available at no cost at certain clinics*	$3,500 - $4,700*<br>**Covered by most insurance plans. Patient responsible for copay or deductible*	$187 - $246*<br>**Covered by most insurance plans. Patient responsible for copay or deductible*
**Time**	10 - 20 minutes	1½ days	½ day
**Frequency**	Every year	Every 10 years	Every 5 years
**Discomfort**	Low	Medium	Medium
**Complication risk**	0	1 in 1000	1 in 7500

SHOW THIS BROCHURE TO YOUR DOCTOR

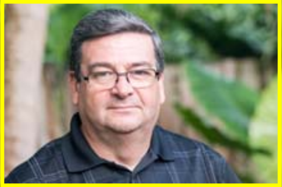

I'm thinking about getting a test but need more information.

I would like to get more information about:

☐ **FOBT**

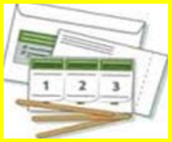

☐ **Colonoscopy**

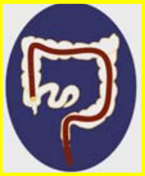

☐ **Sigmoidoscopy**

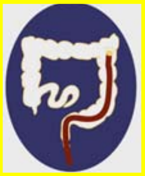

☐ **All three tests**

Show this brochure to your doctor

Show this brochure to your doctor

Dear medical provider:

Your patient recently viewed a video regarding colon cancer screening. By choosing this brochure, your patient has indicated that he/she is thinking about getting a colon cancer screening, but would like more information. He/she has chosen a preferred testing method marked in the front of this brochure. The inside of this brochure contains a comparison table that may be useful as you discuss screening options with your patient.

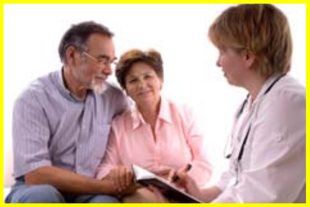

PROJECT CHAT

Linda K. Ko, PhD
Center for Health Communication Intervention (CHealCI)
Fred Hutchinson Cancer Research Center
1100 Fairview Ave. N
Seattle, WA 98019

Phone: **1-855-682-7998**

Email: **earodrig@fhcrc.org**

Web: **http://research.fhcrc.org/chealci/en.html**

Colon cancer screening tests

The **FOBT**, or stool test, is done at home using special cards to determine if the stool sample contains blood.

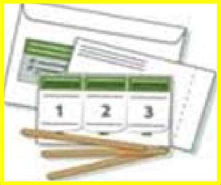

In the **colonoscopy** test, a narrow and illuminated tube is inserted through the rectum and is used to examine the entire colon. It's necessary to take a day off from work and someone has to drive you home after the test

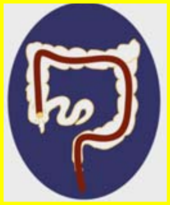

In the **sigmoidoscopy** test, a narrow and illuminated tube is inserted through the rectum and is used to examine the lower portion colon. It's necessary to take the morning off from work and someone has to drive you home after the test.

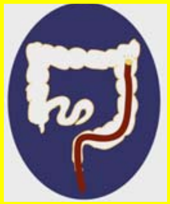

You can benefit by getting a colon cancer screening test, even if you don't have any symptoms

	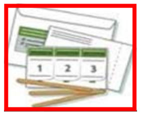 FOBT	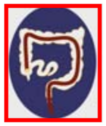 Colonoscopy	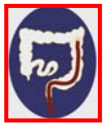 Sigmoidoscopy
**Effectiveness**	Medium	High	High
**Cost**	$5 - 60*<br>**Available at no cost at certain clinics*	$3,500 - $4,700*<br>**Covered by most insurance plans. Patient responsible for copay or deductible*	$187 - $246*<br>**Covered by most insurance plans. Patient responsible for copay or deductible*
**Time**	10 - 20 minutes	1½ days	½ day
**Frequency**	Every year	Every 10 years	Every 5 years
**Discomfort**	Low	Medium	Medium
**Complication risk**	0	1 in 1000	1 in 7500

SHOW THIS BROCHURE TO YOUR DOCTOR

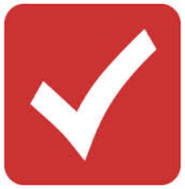

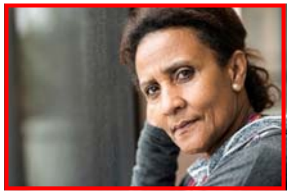

I'm not ready to get a test at this time.

There are 3 tests available, you have options:

FOBT

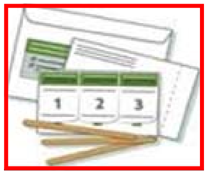

Colonoscopy

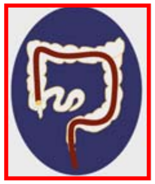

Sigmoidoscopy

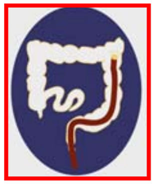

Show this brochure to your doctor

Show this brochure to your doctor

Dear medical provider:

Your patient recently viewed a video regarding colon cancer screening. By choosing this brochure, your patient has indicated that he/she is not ready to be screened for colon cancer. The inside of this brochure contains a comparison table that may be useful as you discuss colorectal cancer screening with your patient.

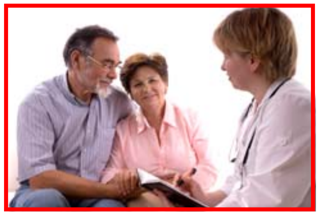

PROJECT CHAT

Linda K. Ko, PhD
Center for Health Communication Intervention (CHealCI)
Fred Hutchinson Cancer Research Center
1100 Fairview Ave. N
Seattle, WA 98019

Phone: **1-855-682-7998**

Email: **earodrig@fhcrc.org**

Web: **http://research.fhcrc.org/chealci/en.html**

Colon cancer screening tests

The **FOBT**, or stool test, is done at home using special cards to determine if the stool sample contains blood.

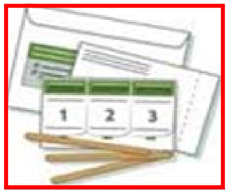

In the **colonoscopy** test, a narrow and illuminated tube is inserted through the rectum and is used to examine <u>the entire colon</u>. It's necessary to take a day off from work and someone has to drive you home after the test.

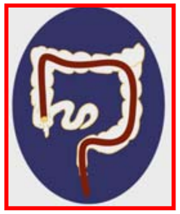

In the **sigmoidoscopy** test, a narrow and illuminated tube is inserted through the rectum and is used to examine <u>the lower portion colon</u>. It's necessary to take the morning off from work and someone has to drive you home after the test.

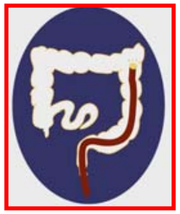

Nos da mucho gusto en saber que usted quiere hacerse una prueba del cáncer de colon

	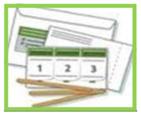 **FOBT**	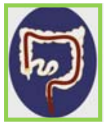 **Colonoscopía**	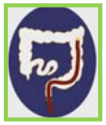 **Sigmoidoscopía**
**Efectividad**	Media	Alta	Alta
**Costo**	$5 - 60*<br>**Disponible sin costo en ciertas clínicas*	$3,500 - $4,700*<br>**Cubierto por la mayoría de seguros médicos. Paciente responsable por co-pago o deducible*	$187 - $246*<br>**Cubierto por la mayoría de seguros médicos. Paciente responsable por co-pago o deducible*
**Tiempo**	10 - 20 minutos	1½ días	½ día
**Frecuencia**	Cada año	Cada 10 años	Cada 5 años
**Incomodidad**	Bajo	Media	Media
**Riesgo a complicaciones**	0	1 en 1000	1 en 7500

MUESTRE ESTE FOLLETO A SU DOCTOR

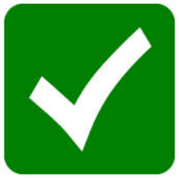 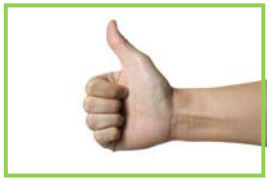

Estoy listo/a para hacerme una prueba.

Preferiría hacerme:

☐ **FOBT** 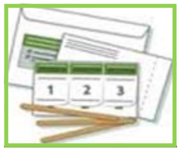

☐ **Colonoscopía** 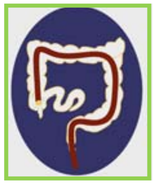

☐ **Sigmoidoscopía** 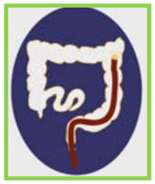

☐ **No tengo preferencia pero quiero hacerme una prueba.**

Muestre este folleto a su doctor

Muestre este folleto a su doctor

Dear medical provider:

Your patient recently viewed a video regarding colon cancer screening. By choosing this brochure, your patient has indicated that he/she is ready to be screened for colon cancer and has chosen a preferred testing method marked in the front of this brochure. The inside of this brochure contains a comparison table that may be useful as your patient proceeds to be screened for colon cancer.

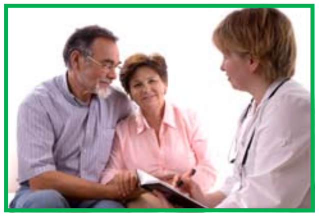

PROJECT CHAT

Linda K. Ko, PhD
Center for Health Communication Intervention (CHealCI)
Fred Hutchinson Cancer Research Center
1100 Fairview Ave. N
Seattle, WA 98019

Phone: **1-855-682-7998**

Email: **earodrig@fhcrc.org**

Web: **http://research.fhcrc.org/chealci/en.html**

Las pruebas para la detección del cáncer de colon

El **FOBT**, o la prueba de la material fecal, se realiza en casa usando tarjetas para determinar si la material fecal contiene sangre.

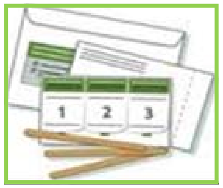

La **colonoscopía** examina el colon entero usando un tubo estrecho e iluminado que se inserta en el recto. Hay que tomar un día libre de trabajo y se necesita a alguien para llevarle a casa.

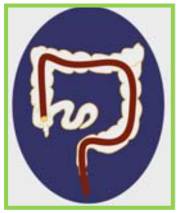

La **sigmoidoscopía** examina la parte inferior del colon usando un tubo estrecho e iluminado que se inserta en el recto. Hay que tomar la mañana libre de trabajo y se necesita a alguien para llevarle a casa.

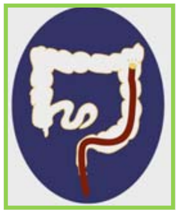

Cuando se haga una prueba de cáncer de colon, se sentirá bien de haberlo hecho por usted y por su familia

	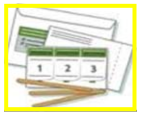 **FOBT**	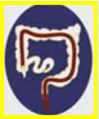 **Colonoscopía**	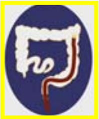 **Sigmoidoscopía**
**Efectividad**	Media	Alta	Alta
**Costo**	$5 - 60*<br>**Disponible sin costo en ciertas clínicas*	$3,500 - $4,700*<br>**Cubierto por la mayoría de seguros médicos. Paciente responsable por co-pago o deducible*	$187 - $246*<br>**Cubierto por la mayoría de seguros médicos. Paciente responsable por co-pago o deducible*
**Tiempo**	10 - 20 minutos	1½ días	½ día
**Frecuencia**	Cada año	Cada 10 años	Cada 5 años
**Incomodidad**	Bajo	Media	Media
**Riesgo a complicaciones**	0	1 en 1000	1 en 7500

MUESTRE ESTE FOLLETO A SU DOCTOR

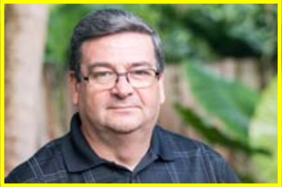

Estoy pensando hacerme una prueba pero necesito más información.

Me gustaría más información sobre:

☐ **FOBT**

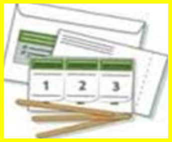

☐ **Colonoscopía**

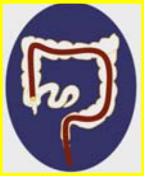

☐ **Sigmoidoscopía**

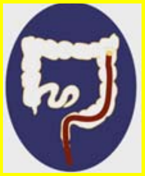

☐ **La tres pruebas**

Muestre este folleto a su doctor

Muestre este folleto a su doctor

Dear medical provider:

Your patient recently viewed a video regarding colon cancer screening. By choosing this brochure, your patient has indicated that he/she is thinking about getting a colon cancer screening, but would like more information. He/she has chosen a preferred testing method marked in the front of this brochure. The inside of this brochure contains a comparison table that may be useful as you discuss screening options with your patient.

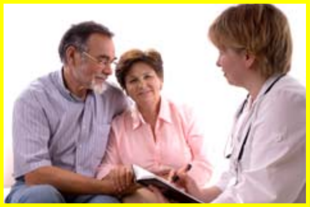

PROJECT CHAT

Linda K. Ko, PhD
Center for Health Communication Intervention (CHealCI)
Fred Hutchinson Cancer Research Center
1100 Fairview Ave. N
Seattle, WA 98019

Phone: **1-855-682-7998**

Email: **earodrig@fhcrc.org**

Web: **http://research.fhcrc.org/chealci/en.html**

Las pruebas para la detección del cáncer de colon

El **FOBT**, o la prueba de la material fecal, se realiza en casa usando tarjetas para determinar si la material fecal contiene sangre.

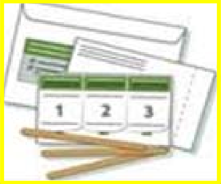

La **colonoscopía** examina <u>el colon entero</u> usando un tubo estrecho e iluminado que se inserta en el recto. Hay que tomar un día libre de trabajo y se necesita a alguien para llevarle a casa.

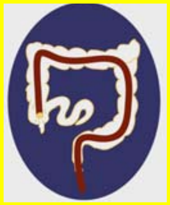

La **sigmoidoscopía** examina <u>la parte inferior del colon</u> usando un tubo estrecho e iluminado que se inserta en el recto. Hay que tomar la mañana libre de trabajo y se necesita a alguien para llevarle a casa.

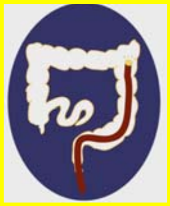

Usted puede beneficiarse haciéndose una prueba del cáncer de colon aunque no tenga síntomas

	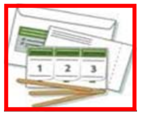 **FOBT**	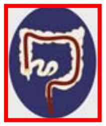 **Colonoscopía**	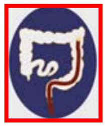 **Sigmoidoscopía**
**Efectividad**	Media	Alta	Alta
**Costo**	$5 - 60*<br>**Disponible sin costo en ciertas clínicas*	$3,500 - $4,700*<br>**Cubierto por la mayoría de seguros médicos. Paciente responsable por co-pago o deducible*	$187 - $246*<br>**Cubierto por la mayoría de seguros médicos. Paciente responsable por co-pago o deducible*
**Tiempo**	10 - 20 minutos	1½ días	½ día
**Frecuencia**	Cada año	Cada 10 años	Cada 5 años
**Incomodidad**	Bajo	Media	Media
**Riesgo a complicaciones**	0	1 en 1000	1 en 7500

MUESTRE ESTE FOLLETO A SU DOCTOR

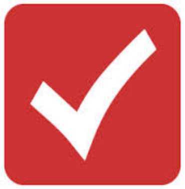
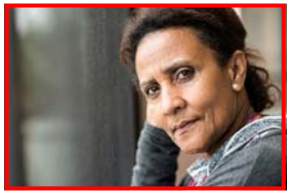

Ahora no estoy listo/a para hacerme un prueba.

Hay tres pruebas disponibles, usted tiene opciones:

FOBT

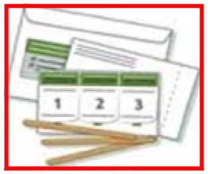

Colonoscopía

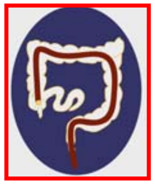

Sigmoidoscopía

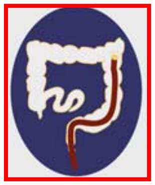

Muestre este folleto a su doctor

Muestre este folleto a su doctor

Dear medical provider:

Your patient recently viewed a video regarding colon cancer screening. By choosing this brochure, your patient has indicated that he/she is not ready to be screened for colon cancer. The inside of this brochure contains a comparison table that may be useful as you discuss colorectal cancer screening with your patient.

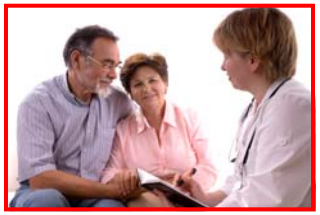

PROJECT CHAT

Linda K. Ko, PhD
Center for Health Communication Intervention (CHealCI)
Fred Hutchinson Cancer Research Center
1100 Fairview Ave. N
Seattle, WA 98019

Phone: **1-855-682-7998**

Email: **earodrig@fhcrc.org**

Web: **http://research.fhcrc.org/chealci/en.html**

Las pruebas para la detección del cáncer de colon

El **FOBT**, o la prueba de la material fecal, se realiza en casa usando tarjetas para determinar si la material fecal contiene sangre.

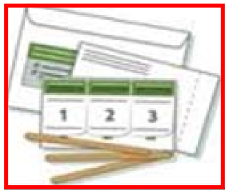

La **colonoscopía** examina el colon entero usando un tubo estrecho e iluminado que se inserta en el recto. Hay que tomar un día libre de trabajo y se necesita a alguien para llevarle a casa.

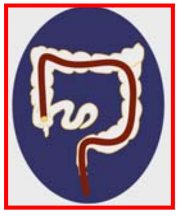

La **sigmoidoscopía** examina la parte inferior del colon usando un tubo estrecho e iluminado que se inserta en el recto. Hay que tomar la mañana libre de trabajo y se necesita a alguien para llevarle a casa.

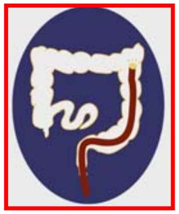

Supplement: Supplementary file 2 — Supplementary Material 2: Appendix B: The intervention brochure. [file 12889_2024_20095_MOESM2_ESM.pdf]
